# Personal

**Published:** 1894-12

**Authors:** 


### Personal.

DR. ARTHUR W. HURD, first assistant physician in the Buffalo State Hospital, has been promoted to be superintendent, *vice* Dr. J. B. Andrews, deceased. It is understood that the board of managers gave to Dr. Hurd its unanimous vote, which has elevated him to this honorable and responsible position.

This, it seems to us, was the only logical course for the managers to pursue, for Dr. Hurd had shown his equipment for the place by many years' service in a subordinate capacity, and had demonstrated his ability to direct its affairs by more than a year's service as acting superintendent. He was also at the head of the list under the Civil Service rules and is medically and every other way fitted for the place. The JOURNAL has much pleasure in extending its congratulations to Dr. Hurd.

---

DR. HERBERT U. WILLIAMS, of Buffalo, who has lately returned from a prolonged visit in Europe, where he has been engaged in study, announces his office at 24 High street. He will give special attention to making autopsies and to microscopical diagnosis, the examination of sputum, urine and blood. Telephone, Tupper 330.

---

DR. FRANK W. ROSS, of Elmira, N. Y., has begun a series of lectures on Electro-therapeutics in the Medical Department of Niagara University. A lecture will be given at the college every first and third Tuesdays of December, January and February.

---

DR. JOSEPH M. MATHEWS, of Louisville, editor of *Mathews's Medical Quarterly*, and the well-known author and teacher, has been appointed president of the Kentucky State Board of Health.

---

DR. CHARLES GILBERT CHADDOCK, of St. Louis, has been appointed associate editor of the *Medical Mirror*.

